# Shell Wounds in the American Navy

**Published:** 1898-06-11

**Authors:** 


### SHELL WOUNDS IN THE AMERICAN NAVY.

Details as to some of the wounds received in the war are beginning to come in. The *Medical Record* states that in the bombardment of San Juan de Porto Rica an eight-inch shell struck the *New York*, exploding at once. One fragment struck a man just behind the ear and emerged at the opposite side of the head. He died almost immediately. Another fragment struck a man in the lower third of the thigh, entering posteriorly and emerging in front. The femur was comminuted. The fragments were resected, and the bone ends wired together by the surgeons on board the *New York*. The same man received a flesh wound in the calf of the other leg from another fragment of the same shell. The third man wounded by this shell received a flesh wound only. A fragment of shell which struck the *Iowa* wounded a marine in the right elbow from behind. The external condyle of the humerus, the head of the radius, and part of the ulna were shattered and carried away. Efforts are being made to save the arm. Several other men received flesh wounds. All the wounds were reported as doing well, and as being aseptic seven days after the receipt of the injuries.

